# “How sweet are your strawberries?”: Predicting sugariness using non-destructive and affordable hardware

**DOI:** 10.3389/fpls.2023.1160645

**Published:** 2023-03-22

**Authors:** Junhan Wen, Thomas Abeel, Mathijs de Weerdt

**Affiliations:** ^1^ Algorithmics Group, Faculty of Electrical Engineering, Mathematics and Computer Science, Delft University of Technology, Delft, Netherlands; ^2^ Delft Bioinformatics Lab, Faculty of Electrical Engineering, Mathematics and Computer Science, Delft University of Technology, Delft, Netherlands

**Keywords:** non-destructive analysis, in-field test, machine learning, computer vision, data fusion, feature selection, total soluble solid, crop management

## Abstract

Global soft fruit supply chains rely on trustworthy descriptions of product quality. However, crucial criteria such as sweetness and firmness cannot be accurately established without destroying the fruit. Since traditional alternatives are subjective assessments by human experts, it is desirable to obtain quality estimations in a consistent and non-destructive manner. The majority of research on fruit quality measurements analyzed fruits in the lab with uniform data collection. However, it is laborious and expensive to scale up to the level of the whole yield. The “harvest-first, analysis-second” method also comes too late to decide to adjust harvesting schedules. In this research, we validated our hypothesis of using in-field data acquirable *via* commodity hardware to obtain acceptable accuracies. The primary instance that the research concerns is the sugariness of strawberries, described by the juice’s total soluble solid (TSS) content (unit: °Brix or Brix). We benchmarked the accuracy of strawberry Brix prediction using convolutional neural networks (CNN), variational autoencoders (VAE), principal component analysis (PCA), kernelized ridge regression (KRR), support vector regression (SVR), and multilayer perceptron (MLP), based on fusions of image data, environmental records, and plant load information, etc. Our results suggest that: (i) models trained by environment and plant load data can perform reliable prediction of aggregated Brix values, with the lowest RMSE at 0.59; (ii) using image data can further supplement the Brix predictions of individual fruits from (i), from 1.27 to as low up to 1.10, but they by themselves are not sufficiently reliable.

## 1 Introduction

Soft fruits such as strawberries, raspberries, blueberries, etc. are popular and profitable fruit varieties. The annual consumption of strawberries in Europe is estimated to be more than 1.2 million tonnes, which leads the market share of horticultural crops (Calleja et al., 2012; Ministry of Foreign Affairs (CBI), 2021a; Ministry of Foreign Affairs (CBI), 2021b). Worldwide production of strawberries is stable with increasing demands and prices and is continuously growing even through the COVID-19 pandemic (Chandler et al., 2012; Bos-Brouwers et al., 2015; Simpson, 2018; Ministry of Foreign Affairs (CBI), 2021b). However, without the protection of hard skins, soft fruits are vulnerable during production and post-harvest activities. This results in significant food waste and economic loss (Fruitteelt, 1991; Food and Agriculture Organization of the United Nations, 2011; 31 Stenmarck et al., 2016). The food loss and waste comprise up to 50% loss along the supply chain in some countries (Macnish, 2012; Kelly et al., 2019), among which the production loss is the majority, which consists of up to 20% (Terry et al., 2011; Porat et al., 2018). It has been estimated that for every ton of food waste, €1,900 of production and processing costs are lost. Moreover, it is argued that 50% of the waste could be edible (Stenmarck et al., 2016).

The nutritional and economic value of crops is influenced by the harvesting strategy. However, subjective assessments and inappropriate maintenance of fruit quality could bring conflicts in logistics planning between suppliers and distributors, which results in even further post-harvest loss (Ramana et al., 1981; Elik et al., 2019). Therefore, early decision-making supports both ecological and economic interests. To make logistic and harvesting decisions as early as possible, it is highly desirable to predict the quality of ready-to-harvest strawberries in the field (Abasi et al., 2018; Corallo et al., 2018; Soosay and Kannusam, 2018; Lezoche et al., 2020).

Multiple variables determine the quality of a strawberry, including maturity, shape, sweetness, and firmness (Montero et al., 1996; Xu and Zhao, 2010; Liu et al., 2014). As the majority of strawberry products are consumed fresh, the taste is the highest priority for most European consumers of strawberries (Chandler et al., 2012; Ministry of Foreign Affairs (CBI), 2021b). Therefore, we narrow our research scope of this paper to concern the interior quality of the fruit, which is not directly told by their appearances: this paper explores the assessment of the level of sweetness of strawberries, which is quantitatively described by total soluble solid (TSS) content in the juice of freshly harvested fruits, using informatics and machine learning (ML) approaches.

Traditionally, the TSS content is measured by a refractometer, quantified by the degree Brix (°Brix or Brix) (Azodanlou et al., 2003). The measurement is expensive in both labor cost and capital because the samples that are sent to destructive measurements can no longer be sold (Gómez et al., 2006; Agulheiro-Santos et al., 2022). To reduce errors and optimize the supply chain, there is a desire for more accurate, quantitative, and non-destructive tools to assess the quality of each fruit (Ventura et al., 1998; Mancini et al., 2020). Therefore, we explore the feasibility of Brix prediction with easily-acquirable data, such that the prediction can be carried out on-site without specific fruit preparation.

Related research has demonstrated the feasibility of applying computer vision (CV) in grading the quality of fruits (Zhang et al., 2016; Liu et al., 2017; Munera et al., 2017; Klinbumrung and Teerachaichayut, 2018) and in assessing specific quality attributes (Montero et al., 1996; Azodanlou et al., 2003; Abeytilakarathna et al., 2013; Vandendriessche et al., 2013). CV and spectral analysis from hyperspectral imaging (HSI) are popular techniques that have often been applied in investigating the intrinsic properties (Amodio et al., 2019; Liu et al., 2019; Gao et al., 2020; Agulheiro-Santos et al., 2022). High prediction accuracy could be was achieved when fruit photos were acquired under a (mostly-)uniform experiment setup (Xu and Zhao, 2010; Nandi et al., 2016; Mancini et al., 2020; Weng et al., 2020; Shao et al., 2021). Such setup requires delicate devices which hinder the applications in a real-world setting and on an enormous number of samples. Moreover, the “harvest first, analysis second” methodology limits the possibility of adjusting the harvest strategy for supply chain optimizations because strawberries stop growing after being harvested. Hence, our study concerns the implication of the fruit’s intrinsic characteristics by its appearance under natural light, when the fruit is still on the plant.

Meanwhile, the micro-climate in the greenhouse and the horticultural treatments strongly influence the harvest quality and pace of growing (Choi et al., 2015; Sim et al., 2020; Díaz-Galián et al., 2021). The temperature, humidity, CO2 level, lighting conditions, and irrigation are proven to be crucial factors (Hidaka et al., 2016; Avsar et al., 2018; Corallo et al., 2018; Muangprathub et al., 2019; Sim et al., 2020). The crop load is also argued to influence the quality of fruits (Verrelst et al., 2013; Belda et al., 2020; 76 Correia et al., 2011). In modern horticulture, environmental data is readily collected by field sensors or climate computers in most greenhouses (Hayashi et al., 2013; Samykanno et al., 2013; Muangprathub et al., 2019; Sim et al., 2020). Nevertheless, these point measurements cannot provide distinctive information to specify the quality of individual fruits. Thus, our research introduces approaches to integrate in-the-wild fruit images with environmental and plant-load data in predicting the Brix values of individual fruits.

By investigating the performances of Brix prediction models, we aim at providing insights in answering two main questions: i) how accurately can the models estimate the Brix values by different sets of inputs? and ii) which data are valuable for training the Brix prediction models? The research contributes from four perspectives: i) we collected and labeled a dataset of strawberry images and quality measurements, using commodity hardware; ii) we designed a conceptual methodology of non-destructive quality estimation; iii) we shaped and implemented our methodology to predict the strawberry sugariness; iv) by comparing the model performances, we suggest how to develop reliable prediction models by CV and ML techniques.

## 2 Materials and methods

### 2.1 Data collection

Data were collected from May 2021 to November 2021. This was carried out on overwintered trays of *Favori* strawberry plants in a greenhouse at the Delphy Improvement Centre B.V. (Delphy) in Bleiswijk, the Netherlands. Strawberries were cultivated in baskets that were hung from the ceiling in the greenhouse. For the plants monitored by the cameras, the harvesting frequency is mostly once per week, or twice per week when the strawberries grow faster in warmer periods. There is exactly one harvest round per day, so we use “from a harvest” to describe the data collected from the same date.

The data collection setup consisted of the following parts: i) static cameras facing the planting baskets to take periodic photos; ii) Brix measurements of the strawberries by the horticulturalists from Delphy; iii) physical labels on the branches to identify the measurement results of a strawberry with its appearance in images; iv) climate sensors to record the environment in the greenhouse and the outside weather; v) plant loads, represented by the average number of *Favori* fruits and/or flowersper unit area; vi) other logs about the plant cultivation.

**Figure 1:**
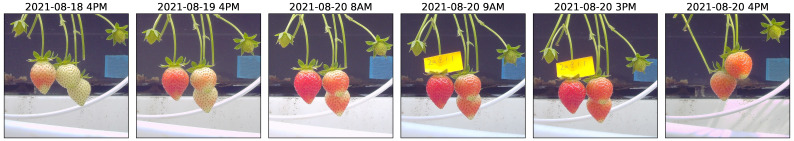
Illustration of the time-lapse images. The same parts of six images are selected. The time stamps of data collection are indicated above the images. According to the images, by 9 am on 2021-08-20, the yellow physical label is stuck onto the branch. The strawberry *20.8.1.1* was harvested between 3 pm and 4 pm of the same day, so the last time when it was observable on images was 3 pm, 2021-08-20.

Representations of individual strawberries were the major inputs to train the Brix prediction models. We considered image data because they are objective and distinct. The images were collected hourly with a time-lapse setting. The same sections of six example images are shown in **Figure 1**. As is shown in the figures, we stuck a yellow label to indicate the ID of a strawberry a few hours before the harvest

(namely the “ID label”), such that the strawberry’s appearance in the images can be connected to the measurement results. The measurement data that are assigned to identified strawberries are called the “connected measurements” in the following text.

Based on previous research on influencing factors of strawberry qualities (Chen et al., 2011; Correia et al., 2011; Avsar et al., 2018) and the expertise of our collaborating horticulturalists, temperature, humidity, radiation level, CO2 density, and relevant plant treatment records (additional lighting, watering were all considered as the environment data. The number of fruits and/or flowers per unit area was counted weekly and noted as the “plant load”. Both the environment and plant load data were collected by Delphy.

The strawberries with the ID labels were stored separately. On the same day of the harvest, researchers from Delphy measured the Brix value and the firmness of those strawberries, with a refractometer and a penetrometer respectively. The size category is defined by a ring test, and the ripeness level is evaluated according to the experience of the greenhouse researchers.

### 2.2 Methodology of experiment implementations

We segmented the strawberries from the in-field images, such that only the pixels that describe the sample strawberry were analyzed. We trained a Mask R-CNN model (He et al., 2017) with a ResNet101 backbone for semantic segmentation. We used the Detectron2 platform (Wu et al., 2019) to build the model. The ResNet101 backbone was pre-trained on the *ImageNet* dataset. We resized the image segments to 200*200*3 pixels. They were the raw inputs for Brix prediction and feature extraction in the pa, the *image-with-env experiment*, and the *image-with-Brix experiment*. We considered only the last available observations, e.g. the strawberry segment from the 5 image in **Figure 1**. In this way, we limited the quality changes between when it was in the image and when it was measured. We also normalized the colors of the images to reduce the distraction from the changing lighting conditions during the day by applying elastic-net regressions at the red, green, and blue channels respectively.

To analyze the images in the *image-only experiment*, we built Convolutional Neural Networks (CNNs) and Variational Auto-Encoders (VAEs) to analyze and encode the image segments of individual strawberries with Multi-Layer Perceptrons (MLPs). The models were either trained from scratch or with weights pre-trained by other popular datasets such as the ImageNet (Deng et al., 2009). Details of model architectures can be found in the supplementary materials. We also introduced principal component analysis (PCA) in the experiments for feature dimensionality reduction and model regularization (Geladi et al., 1989; Shafizadeh-Moghadam, 2021). By taking the largest differences among the pixel data, PCA helps to exclude disturbance from the shared information of strawberry images to some extent. Hereafter, we use the word “encode” to represent the process of dimensionality reduction by the encoder parts of the VAEs and/or PCA. We use “attribute” to describe the content of information that our model concerns. “Feature” or “input” represents what goes directly to the models, such as information from the latent space of the VAEs and/or after PCA.

We trained the CNNs, MLPs, the predictor part of the VAEs, and the PCA models by the strawberry observations with connected measurements, which are 178 out of 304 Brix measurements. We trained the encoder and decoder parts of the VAEs by all the segmentation outputs of the Mask R-CNN model. Hence, this dataset includes images that were taken over the life cycles and of more strawberries. The *image-only experiment* and the *image-with-env experiment* applied the same encoders.

We designed the *env-only experiment* to analyze the relationship between the environment data and the Brix. We used rolling averages of the environment data over different periods. Since the environment data does not include specific information about individual strawberries, we took all of the 304 Brix measurements into account and grouped them by each harvest. They are called the “aggregated Brix”. The reliability of the aggregated Brix could also be better ensured by introducing more sample measurements. We not only trained machine learning models to predict the value expectation, but also the standard deviation (std.) and the percentiles from 10% to 90% (with intervals of 10%). The representations of the Brix distribution were considered in supporting further experiments of individual Brix prediction.

Since the amount of data points was reduced to the same as the days of harvests after the aggregation, the volume of the dataset became too small to support the training of deep neural networks. Hence, we applied linear regression (LR), support vector regression (SVR), and kernelized ridge regression (KRR) models. In addition, leave-one-out experiments were considered to enlarge the training sets of the *env-only experiment*. That means we split only one data point as the validation set in each experiment run, instead of proportionally splitting. Under this setting, we ensured all the data was used once in performance validation so that we could get a predicted value at every data point. The performance of individual Brix prediction in the *env-only experiment* is discussed based on the results from the leave-one-out experiments, by considering the predicted value expectation as the Brix predictions of all harvests on the same day.

In the *image-with-env experiment*, we stacked the features of images and the environment data according to the object strawberries to train models. By the encoder parts of the VAEs and the PCAs fitting to the training set, we encoded the images to image features. We trained the models of the *image-with-Brix experiment* by the same image features but with the outputs from the *env-only experiment*– predictions of the mean, std., and percentiles, etc. We established four neural network architectures to fit the various size of features in both the *image-with-env experiment* and the xpd, including three three-layer MLPs and one four-layer MLP.

We used the Keras library (Chollet et al., 2015) to build and train the CNNs, VAEs, and MLPs in the experiments. All model training used the Adam optimizer (beta1 = 0.9, beta2 = 0.999) and a learning rate of 0.0003. We considered random rotation, mirroring, and flipping to augment the image data. When training the VAE, we also considered random scaling up to ±10%. We used the Scikit-Learn library (Pedregosa et al., 2011) to conduct PCA and to construct LR, SVR, and KRR models in the *env-only experiment*. The KRR used polynomial kernels of degrees up to 3 and penalty terms of 1 and 10. These are all state-of-the-art implementations in data analytics.

For all experiments except with specific definitions, we split the data into 7:1:2 for training: testing: performance validation. We run each experiment 15 times with a fixed series of data splits. All the deep learning models were trained on a Geforce GTX 1080 GPU under a maximum of 300 epochs.

## 3 Results

This chapter describes our research findings in four steps: i) the exploration of the dataset that we collected; ii) our conceptual methodology of designing the experiments; iii) the model performance of each series of experiments respectively; iv) two influencing feature selections: whether to use the plant load data or not and which image encoder to choose. The last section gives a comparison among the experiment series and states our suggestions for developing a reliable Brix prediction model.

### 3.1 An integrated dataset describing the growth and harvest quality of strawberries

In order to predict Brix from non-destructive in-field data, we collected observations of the fruits and related environmental records in a greenhouse. The observations were in the form of images, and the environmental records are time-series and single-value measurements. All relevant data were linked with the observations of individual fruits. As such, we could implement machine learning techniques to discover the mapping from the collected data to the Brix values.

**Figure 2:**
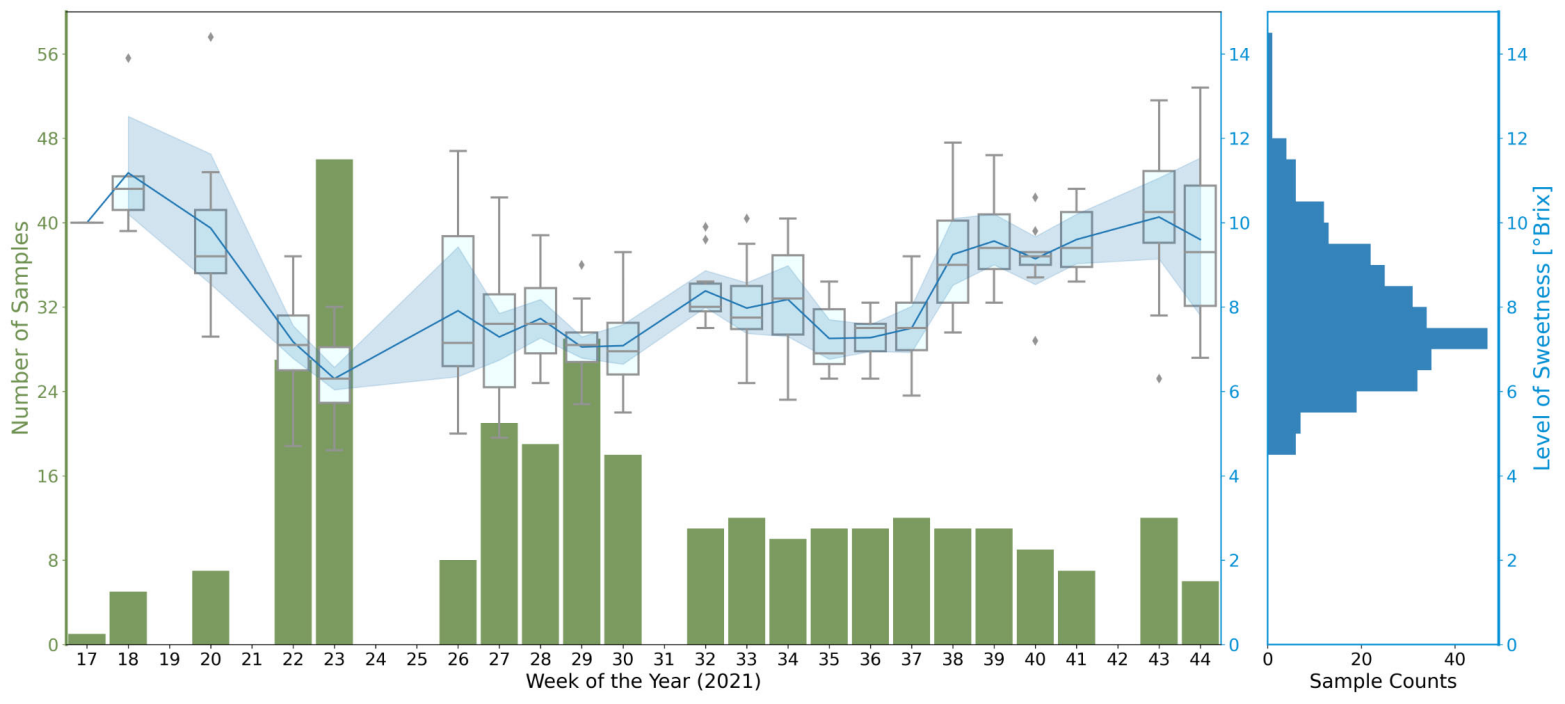
Statistics of the Brix measurements, grouped by harvests per week. On the left, the x-axis indicates the calendar week number of the harvests. The green y-axis presents the number of tested samples. The blue line and its contour indicate the averaged Brix value and the standard deviation (std.) of the measurements of the week respectively. The box plots illustrate the distribution of the measurement for the week. On the right, the histogram gives an overview of the distribution of all Brix measurements in 2021.

From April 2021 to November 2021, we recorded the growth of strawberries by 13,400 images from three RGB cameras and collected environmental records during this period. We measured the Brix of 304 ready-to-harvest strawberries, which were selected from 28harvests in 22 weeks. The overall statistics of the measurement data set are shown in **Figure 2**. According to the box plots and the line plot, the Brix at each harvest usually has a median value lower than the mean. It is implied that using the average sample measurements to estimate the Brix of every fruit has a higher probability to overestimate the quality.

The environmental records during the data collection period were archived hourly and were grouped by rolling averaging over periods. As a preliminary analysis, we computed the correlations of the environmental data under different averaging periods and the aggregated Brix values of each harvest. The results indicate a strong correlation between temperatures (measured on the leaves, plants, and in the air), radiation levels, watering, and cyclic lighting strengths with the mean Brix of each harvest. The correlations of the Brix with humidity and CO2 density are weaker. Details are shown in **Figure S2** in **Supplementary Materials**.

### 3.2 Conceptual experiment design

**Figure 3:**
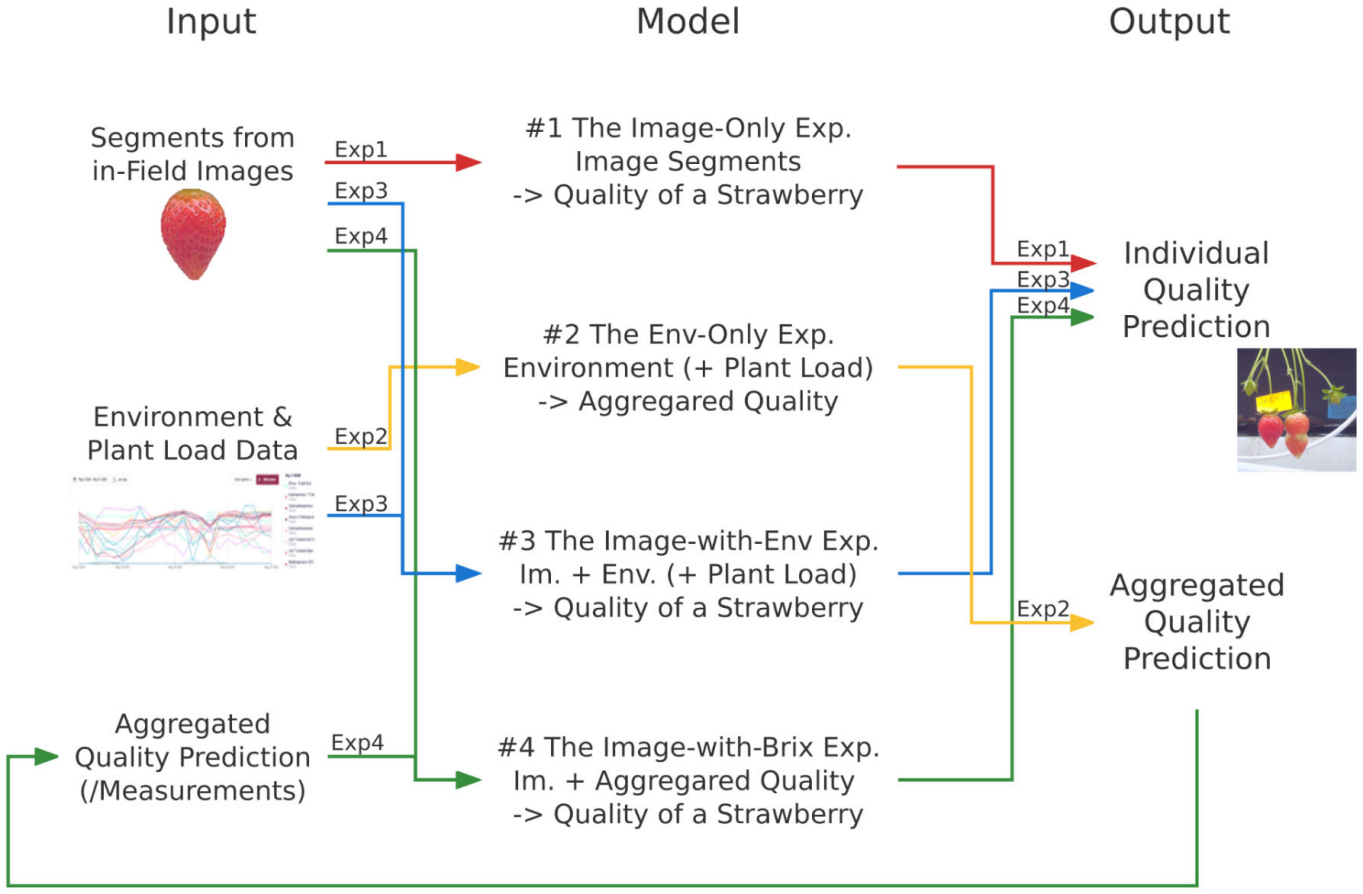
The methodology of the four experiment series in this research. They are described by the data flow, consisting of the input attributes, the output objects, and the models to map the corresponding inputs and outputs. The line colors and the short notes indicate different experiment series: red represents the *image-only experiment*(**“**Exp1**”**), yellow is for the *env-only experiment*(**“**Exp2**”**), blue is for the *image-with-env experiment*(**“**Exp3**”**), and green is for the *image-with-Brix experiment*(**“**Exp4**”**). All the models are evaluated by comparing the outputs with the ground truth.

We designed four series of experiments to study the effectiveness of using these data, shown in **Figure 3**: we first analyzed whether the images (section 3.3) or the environment data (section 3.4) could work alone in Brix prediction, and then we considered two ways of data fusion (section 3.5).

In the *image-only experiment*, the Brix prediction model was trained solely by the images of strawberries. We considered both supervised learning (SL) and semi-supervised (SSL) in training the models in this experiment series. A challenge in this experiment was that the inclusion of non-relevant pixel data lowered the learning process and even reduced the prediction accuracy. To reduce this effect, some of the models were accompanied by additional regularization procedures, such as conducting principal component analysis (PCA) on the training dataset and using the principal components as the features for learning.

We considered environmental records and/or plant loads as the input in the *env-only experiment*. Together we call these the environment data. In the primary step, we conducted correlation analysis to classify the importance of each sort of attribute and to define sets of features. Since the environment data cannot express information about individual strawberries, we trained regression models to predict the expectation and the distribution of Brix value aggregations of each harvest.

We established the *image-with-env experiment* and the *image-with-Brix experiment* respectively as two ways of integrating the image data and environmental records in training. We encoded the image of each strawberry to comprise the image features. These features were combined directly with the environmental records to train the neural networks in the *image-with-env experiment*. We considered the image features and the aggregated Brix predictions from the *env-only experiment* as the inputs in the *image-with-Brix experiment*. The setup was chosen based on two assumptions: i) the predictions from the *env-only experiment* are good indications of the overall quality of harvests; ii) compared to predicting the absolute Brix, the appearance information might be more helpful in terms of estimating the relative position out of value distribution of Brix.

We set up two exam baselines to evaluate the experiment outcomes. Firstly, we used the average value of all the Brix measurements as the expectation of the *Favori* species. It represents the empirical Brix value that members of the soft fruit supply chain usually believe, so it is named the *empirical baseline*. It is the baseline of this Brix prediction study. Secondly, we considered the average Brix of each harvest as the expected value. As it represents the traditional way of sugariness assessment, which is anticipated by sample measurements, it is called the *conventional baseline*. According to the experiment setup, the *conventional baseline* is essentially the optimal situation of models from the *env-only experiment*.

We used root mean squared error (RMSE) and mean absolute error (MAE) to represent the model accuracy. The RMSE is regarded as the main indicator of model performance. It gives increasingly more punishments if the predicted value is further from the ground truth. After running the experiments over different dataset splits, we used the standard deviation of the RMSEs (RMSE-std.) to indicate the robustness of model performances. The coefficient of determination (also called the R2 score) is considered a quantitative assessment of the level of model fitting. It is the proportion of the variation in the dependent variable, i.e. the individual or the aggregated Brix in this case, that is predictable from the input data. Higher R2 scores indicate better correlations between the inputs and outputs in the mapping.

### 3.3 Practical Brix prediction models cannot be trained with images alone

By the *image-only experiment*, we inspect the feasibility to train a Brix predictor with only images. We trained CNNs from scratch, with transfer learning (TL), and with semi-supervised learning (SSL) methods. The best-performing model of the entire experiment series has an averaged RMSE of ca. 1.33 over different validation splits.

**Figure 4:**
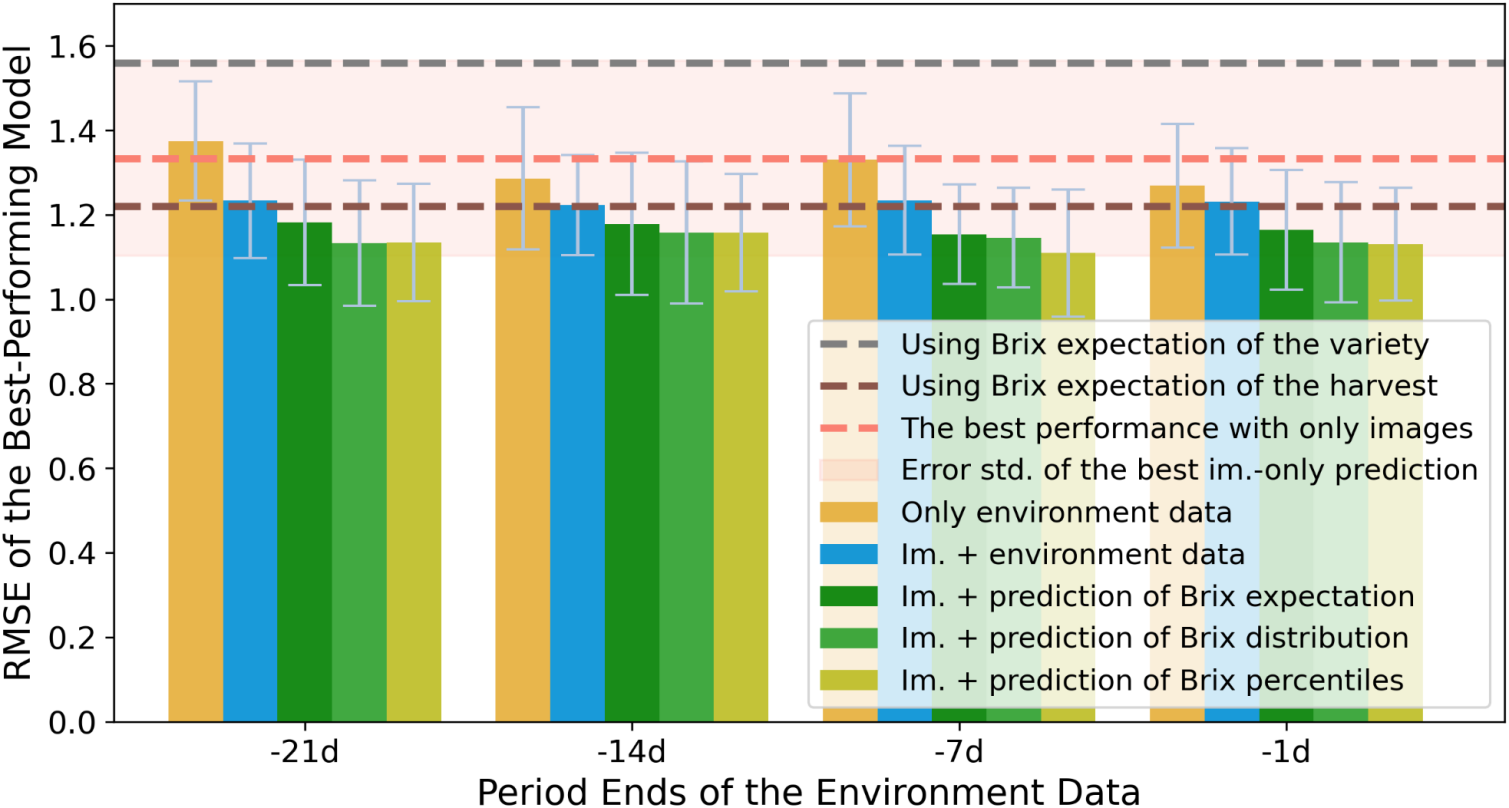
Performance comparison of Brix prediction accuracies among the four experiment sets, using RMSE as an indicator. The error bars indicate the standard deviation of RMSEs (RMSE-std) across different splits of validation sets. The models are grouped by the ending point of the periods of the environmental records. The y-axis shows the minimum RMSE of models from the same group. The colors indicate the input attributes of the experiment sets. The best performance of models using only image data is presented by a horizontal line. The contour around it indicates the corresponding RMSE-std. The horizontal line in gray and brown indicates the two benchmarks that are mentioned in the methodology section.

As the horizontal lines in **Figure 4** indicate, the selected model outperforms *empirical baseline*, while it is slightly worse than *conventional baseline*. It is implied that the appearances of strawberries provide hints of the Brix to a limited extent, whereas the time of harvest has more predictive power. We hence conducted further experiments to unravel the other attributes for Brix prediction.

Among the experiment results, we noticed that the involvement of feature dimensionality reduction facilitates the model performance. A possible mechanism would be that a large proportion of overlapping features were condensed in the latent space of VAEs or the orthonormal bases of PCA (Goharian et al., 2007). Meanwhile, the model fitting might also be regularized with the help of PCA, particularly when the model was trained with a small data set in our situation (Geladi et al., 1989; Delac et al., 2005). These findings also motivated us to encode the images in the data fusion steps of further experiments.

### 3.4 Models reveal significant dependencies of aggregated Brix on environment data

#### 3.4.1 The performance in predicting aggregated values

In the *env-only experiment*, we trained LR, SVR, and KRR models to assess how well the collective Brix value can be predicted with only the environment data. When aggregating the data points, overfitting was an indispensable issue. Particularly, when the data are very few whilst the inputs have a large dimension. To assess the level of model-fitting, we calculated the R2 score of models using different subsets of features, hyper-parameters, and train-test splits to predict the representations of value aggregations on the testing data set. When we grouped the scores by the algorithms of models to evaluate the level of model determination, we found more than half of the LR models have a negative R2 score, which indicates that simple linear models cannot fit this mapping. With a stronger regularizer, or with higher outlier flexibility, the R2 scores of KRR (alpha=10) and SVR models are more condensed to 0.5-0.6. The generally higher R2 scores also indicate they are more practical models in tackling this circumstance.

#### 3.4.2 The performance in predicting individual values

To make the results comparable, the predictions of the averaged Brix were regarded as the estimation of all the strawberry measurements at each harvest. The RMSEs were hence calculated on the same validation splits as the other experiment sets take. **Figure 4** compares the effectiveness of using various periods of environment data with other experiments, of which the time spans are grouped by the ending time.

As the bars in **Figure 4** demonstrate, when models use features from the periods closer to the harvest time, they obtain lower and less diverged RMSEs in general. The RMSE-std of the models in the *env-only experiment* is lower than the best-performing model from the *image-only experiment*. The result argues that even using only the environment data in Brix prediction could train more reliable and stable models. Hence, it is strongly suggested to involve the environment data in training further comprehensive models.

### 3.5 Images give the power to perform individual prediction with environment data

Results from the *env-only experiment* indicate that we need specific information to distinguish fruit-to-fruit differences from each harvest. Since the environment data are all point measurements, we encoded the images into 200, 50, 10, and 5 features by four combinations of VAEs and PCA respectively to fit the dimension differences between the two types of data. The *image-with-env experiment* and the *image-with-Brix experiment* introduce two ways of fusing the image feature and environment data.

#### 3.5.1 Combining image features with direct environmental information

The *image-with-env experiment* straightforwardly combined the two types of data to train the MLPs for the individual Brix prediction. Unsurprisingly, the lowest RMSEs from all the groups outperformed the best models from the *image-only experiment* and the *env-only experiment*, as is illustrated in **Figure 4**.

Curiously, the performance difference caused by the collection time span of environment data was remarkably reduced in this experiment. A possible reason would be that the MLPs also learn the trend of changes within the time-series data – such that the performance did not reduce as much as in the *env-only experiment*. Meanwhile, the nonlinearity and regularization performed by the neural network also ensured the robustness of the model performances.

#### 3.5.2 Combining image features with predicted Brix distribution of a harvest

The fourth experiment, the *image-with-Brix experiment*, allows us to explore another way of integrating the knowledge from the two sorts of data: to use the image features to predict the relative quality within the distribution of Brix values. We used the predictions of Brix aggregations^1^ from the leave-one-out experiments from the *env-only experiment*. Among all the experiment series, the models from the *image-with-Brix experiment* resulted in the lowest RMSEs, as illustrated in **Figure 4**. Among the different features of the aggregated Brix, models that were trained by Brix percentiles slightly outperform the models that assumed a Gaussian-distribution fit, i.e. using the mean and std. as inputs.

### 3.6 Plant load is crucial as part of the indirect environmental information

**Figure 5:**
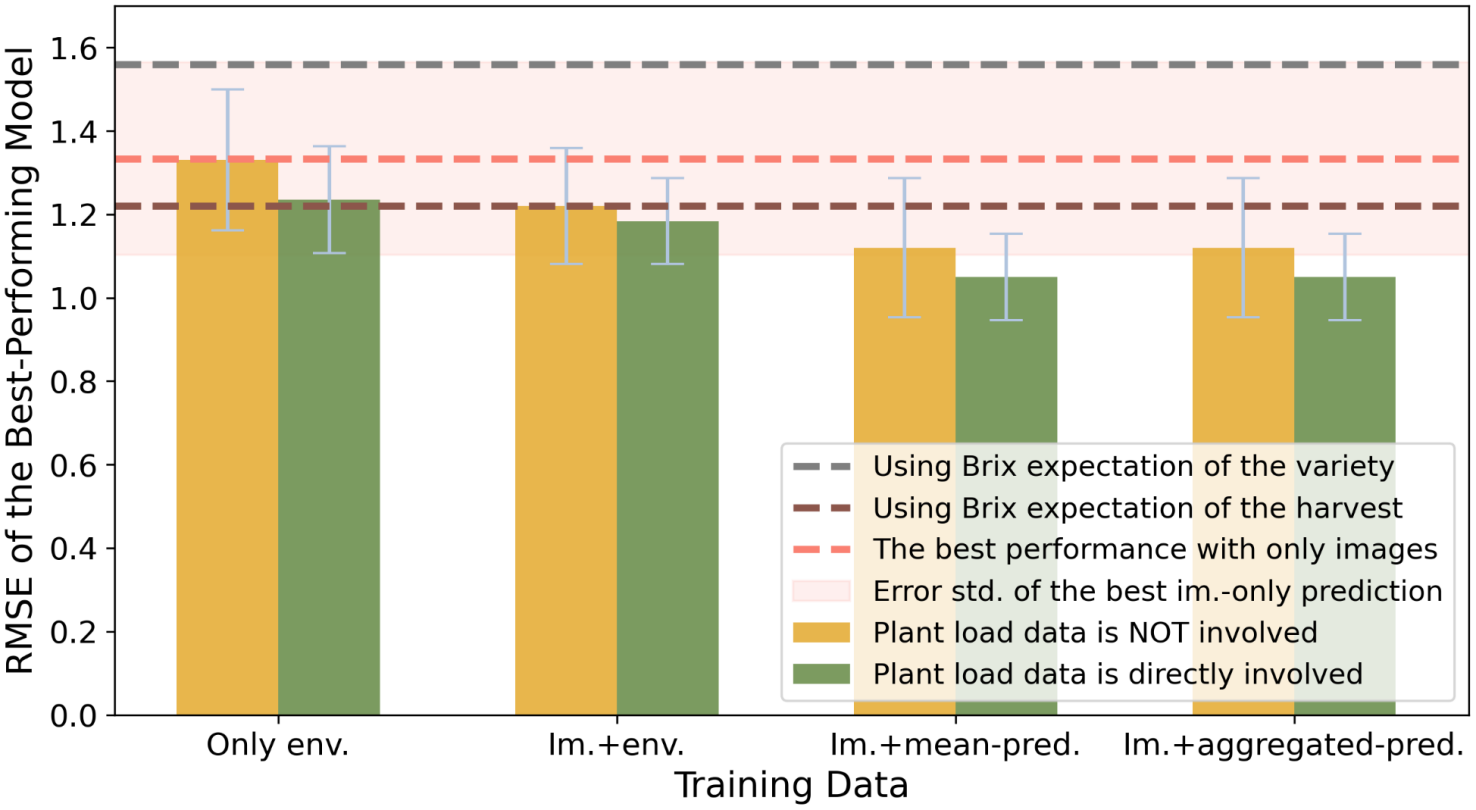
Performance comparison of Brix prediction using different attributes of environmental information, using RMSE as an accuracy indicator. The colors indicate the involvement of the plant load data. The y-values indicate the minimum RMSEs of models from the same group.

As is illustrated in **Figure 5**, introducing the plant load as part of the input attributes has a positive effect on the model performances, which is more outstanding on the models from the *env-only experiment*. In the *image-with-env experiment*, the upper limit of model accuracy was slightly improved. But more importantly, there were notable decreases in the std. of RMSEs over different data splits. Both changes were limited in the *image-with-Brix experiment*. In all, we suggest that plant load is a crucial feature when the raw environmental information comprises the input data.

Moreover, since our plant load data was averaged over different branches of strawberries, they did not directly reflect the division of nutrition on the camera-monitored plants as the literature suggests. Hence, we suppose that the data could reveal the general influence of the growing environment on strawberries in this greenhouse compartment in an indirect and deferred way.

### 3.7 Image encoders have a noteworthy influence on the model performances

**Figure 6:**
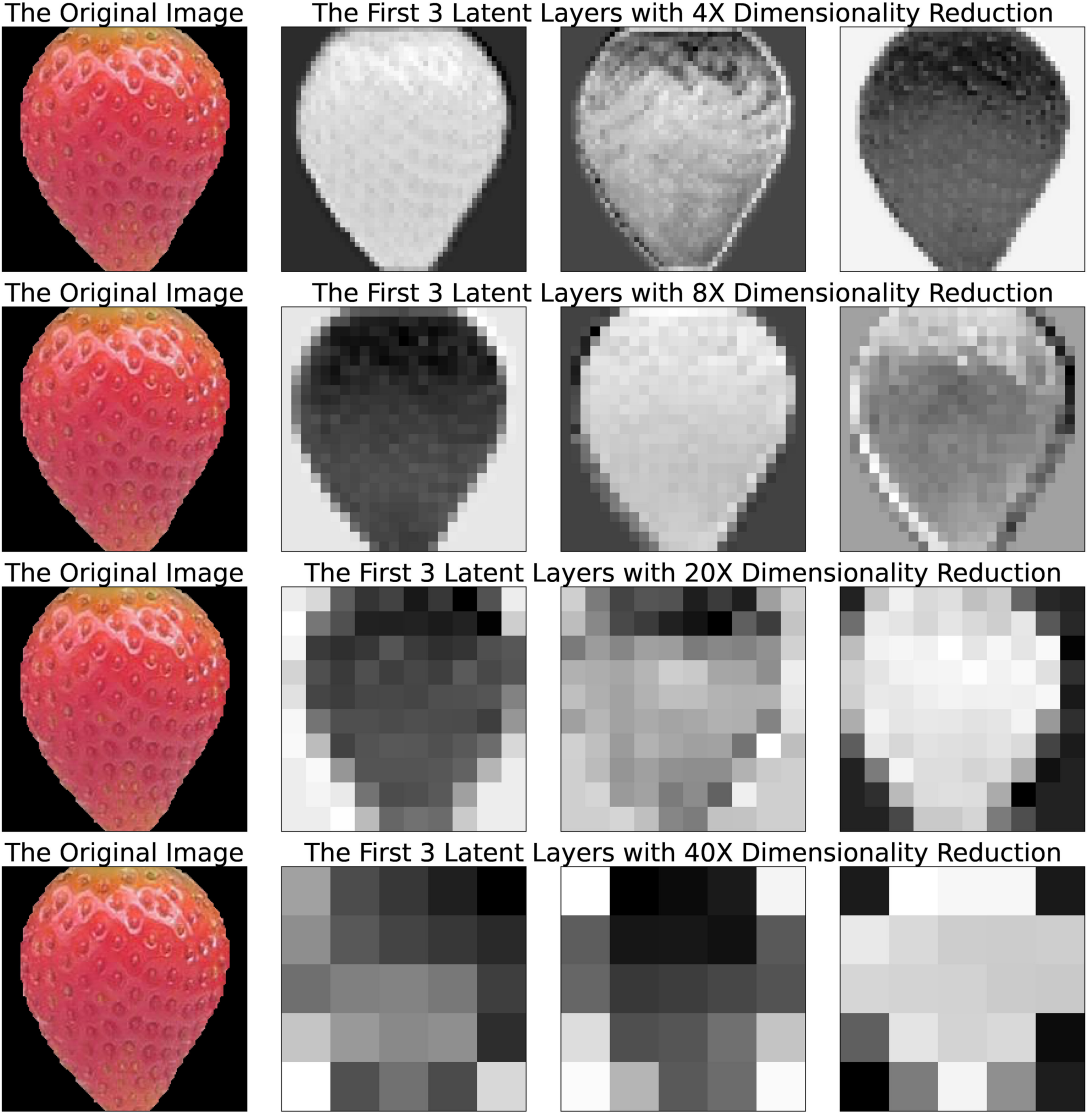
Examples of an image segment and its latent features from the four VAEs, plotted in a monologue style. The first column is the original image segment uniformed into a size of 200x200 pixels. The segment background is saved as black and transparent pixels. The level of dimensionality reduction from each encoder is shown on top of the latent space illustrations.

**Figure 7:**
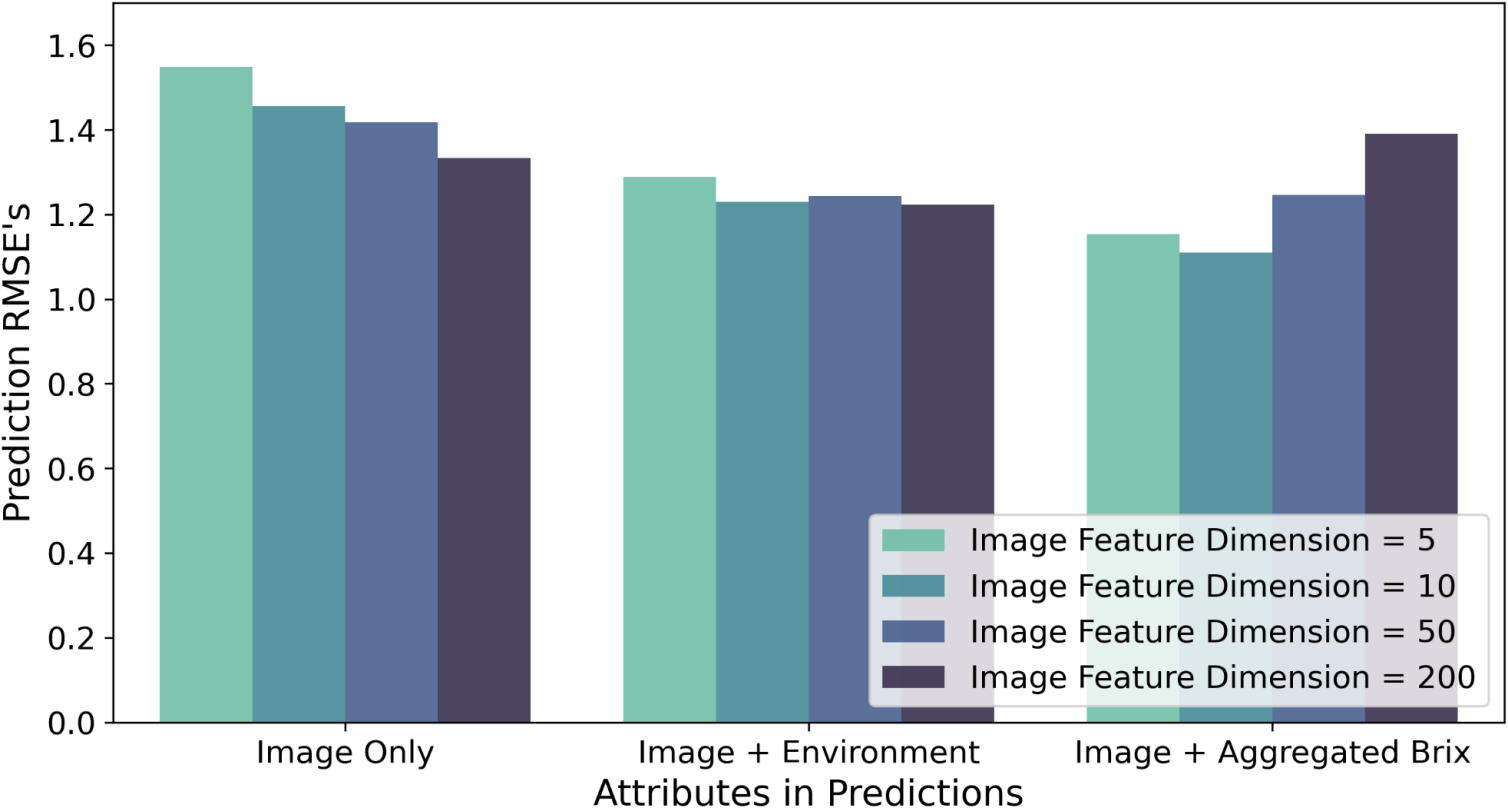
Performance comparison of Brix prediction using different image encoders, using RMSE as an accuracy indicator. The x-axis indicates the input attributes of the experiment sets. The colors indicate the dimensionality of the image features involved in the experiments. The y-values show the minimum RMSEs of all models from the same group.

The best-performing models of each family are considered in the previous result discussions. However, the number of image features also influenced the model accuracy. The information from different latent spaces is illustrated in **Figure 6**. **Figure 7** discusses the effects when the image features are utilized with different representations of environment data. When we used only the images in the prediction, it is still important to keep as many features as possible. Referring to the illustrations in **Figure 6**, it is indicated that considering the texture and the shape of strawberries could have a positive influence on the intrinsic quality representation. When using image features together with the raw environment data, we cannot see much difference in the best performances. Nevertheless, we observe an increase in the RMSEs when using larger dimensions of image features with the aggregated Brix. Overall, it is suggested that similar dimensions of image features and the other source of data could generally achieve better RMSEs.

## 4 Discussion

With this paper, we propose and evaluate a practical methodology for estimating the sugariness of individual strawberries, starting from planning the data collection setups. This approach uses affordable devices to collect relevant observations in the field and does not require harvesting or destroying the fruit. The experiment results demonstrate that it is feasible to anticipate the quality of strawberries when they are still growing. Such information could support the decision-making of harvesting and supply-chain strategies of greenhouse managers.

According to **Figure 4** and **Table 1**, the models using image features with aggregated Brix information are the optimal choices among all the attribute combinations. The models could reduce the RMSE by up to 28.8% and 18.9% from the *empirical baseline* and the *conventional baseline* respectively. Compare to the image data, the environmental information has shown to be more relevant for the models to learn from, yet they lack the capability to tell fruit-to-fruit variances. Compared to using data from a sole source, a mixed-use of both could lead to an accuracy improvement of 10.0% and 6.2%, respectively.

**Table 1:** Detailed accuracy indicators of the best-performing models using different sets of input attributes.

Image Feature	Env. Data		Plant Load		Brix Agg.	MAE	RMSE	RMSE-std.
Included	In Agg. Pred.		In Agg. Pred		Percentiles	0.81	1.10	0.158
Included	In Agg. Pred.		In Agg. Pred		Mean + std.	0.86	1.12	0.139
Included	In Agg. Pred.		In Agg. Pred		Mean	0.90	1.15	0.118
Included	Included		Included		N/A	0.90	1.18	0.103
Included	Included		Not included		N/A	0.90	1.22	0.119
*the conventional baseline*						*0.91*	*1.22*	*0.151*
N/A	Included		Included		N/A	0.96	1.24	0.128
N/A	Included		Not included		N/A	1.00	1.27	0.146
Included	Included		Included		N/A	1.04	1.32	0.134
Included	Not included		Not included		N/A	1.00	1.33	0.189
*the empirical baseline*						*1.21*	*1.56*	*0.312*

The models are ranked according to the **“**RMSE**”** column. the *empirical baseline* is calculated by using the Brix expectation of the strawberry variety as all the predicted values. the *conventional baseline* is calculated by taking the average Brix of each harvest as the individual predictions. The MAE and RMSE of all models and benchmarks are calculated by averaging over 15 random validation splits. The std. of the RMSE on each validation split is presented in the **“**RMSE-std**”** column.

Compared to other research in the field, we included multiple types of data to build machine-learning models. Our models show competitive performances in the sweetness prediction of strawberries [RMSE 1.2 from Sun et al. (2017), MSE 0.95 from Cho et al. (2019)] while using in-field data collected more easily-acquired devices. On top of that, the dataset that we collected for pursuing this research is also useful for more research in this field.

In the above-mentioned experiments, we performed all the procedures step-by-step, yet we see the possibility to exploit higher levels of model integration. Nevertheless, as state-of-the-art computer vision technologies allow detection models to be faster and more portable, expanding the capability of real-time assessments of fruit quality could also be an interesting topic.

The research primarily studies in-field and non-destructive data that are worth to be considered in training Brix prediction models. The images, which the prediction models were trained with, are essentially a subset of the time-lapse image dataset. With the entire dataset, further research is suggested to include temporal information for refining the quality prediction models. It is also an interesting topic to explore the practicability of using earlier images in forecasting future Brix values.

Our results suggest that environmental information plays a vital role in training a reliable model. Particularly, the environmental information from up to fourteen days before the harvest is crucial to ensure the model’s accuracy. Nevertheless, we did not discuss the detailed influence of specific sources of climate data on our model accuracies. It is therefore recommended to conduct subsequent studies on the effectiveness of learning with different environmental factors.

## Data availability statement

The original contributions presented in the study are included in the article/**Supplementary Material**. Further inquiries can be directed to the corresponding author. The datasets that are collected for this study can be found in the 4TU Data Repository: doi:10.4121/21864590.

## Author contributions

JW confirms her contribution to the paper as follows: research conception and design, data (pre-) processing, analysis and interpretation of results, and manuscript preparation. All the work was done under the supervision of MW and TA. All authors reviewed the results and approved the final version of the manuscript. All authors agree to be accountable for all aspects of the work in ensuring that questions related to the accuracy or integrity of any part of the work are appropriately investigated and resolved.

## Footnotes

1 To limit the variables, we took only results from the KRR model with alpha=10 and polynomial degree=3.
