# Supplementary material for: “How sweet are your strawberries?”: Predicting sugariness using non-destructive and affordable hardware
Source: Front Plant Sci. 2023 Mar 22;14:1160645. doi: 10.3389/fpls.2023.1160645 (PMC10075323; doi:10.3389/fpls.2023.1160645)
Supplement: Supplementary file 1 [file Presentation_1.pdf]

Supplementary Material

"How sweet are your strawberries?": predicting sugariness using non-destructive and affordable hardware

1 PRACTICAL DETAILS OF DATA COLLECTION

1.1 Camera Settings

We used cameras from MAPIR®to collect the time-lapse images. We used *Survey3N - Visible Light RGB* for RGB image collection and *Survey3N Camera - Orange+Cyan+NIR (OCN, NDVI)* for OCN images. All cameras had the same settings as we show in Table S1. We set the shutter speed, ISO, and white balance into the auto to maintain an optimal state of each image. No extra exposure was added. We take the neutral setting of color presentation, contrast, and sharpness.

Table S1. Settings of the data collection cameras. Hereby all the changeable settings of the cameras are listed. There is no difference when setting the RGB or the OCN cameras.

Item	Setting
Shutter	Auto
ISO	Auto
White Balance	Auto
Exposure	0
Metering	Centre
Color	Normal
Contrast	Medium
Sharpness	Medium

1.2 Environment Data

Sets of sensors monitored various places in the greenhouse or in the neighborhood at a 5-minute frequency. The data were then averaged by hours to reduce influences from sensor failures. The rainy condition at the location of the greenhouse was recorded as Boolean values, i.e. only 'rained or not' was noted. Additional treatments such as using flowering bulbs and irrigation were logged hourly by greenhouse managers.

Specific dates of measurement data collection are shown in Figure S1.

2 FEATURE GROUPING AND SELECTION

As a preliminary analysis, we computed the correlations of the environmental records and the average Brix of each harvest. We considered the environmental records from the week before the harvest. The results in Figure S2 indicate a strong correlation between temperatures (from the surface, leaves, plants, outdoor, etc.), radiations, watering, and cyclic lighting with the Brix expectation.

[1] "xxx" refers to names of specific (known) locations in the greenhouse

[2] Due to sensor failure during summer, the outdoor PAR value is not considered in further experiments.

[3] Due to errors in the log, the pH values of irrigation are not considered in further experiments.

[4] We know from experience that the automatic measurements can differ from the manual measurements our growing manager takes by hand. Please be aware of that when mentioning the pH anywhere in the results

Table S2. Attributes of Growing Environmental Records. The columns indicate the higher-level groups. The content of each cell is formatted as "abbreviation: explanation". Sensors were installed both inside and outside the greenhouse compartment. All the records were collected every 5 minutes, except the plant load, which is counted every calendar week.

Abbreviation	Content
INDOOR TEMPERATURE	
PT-xxx [1]	Plant Temperature at specific locations
LT-xxx	Leaf Temperature (LT) at specific locations
ST-xxx	Substrate Temperature (ST) at specific locations
GT-afd	Ground Temperature (GT) of the greenhouse compartment
VT-afd	Ventilation Temperature (VT) of the greenhouse compartment
INDOOR HUMIDITY	
AH-afd	Absolute Humidity (AH)
RH-afd	Relative Humidity (RH)
RADIATION	
RDAN	Net radiation
PAR	Photosynthetically Active Radiation (PAR) of the compartment
PAR-xxx	PAR at specific locations
INDOOR MICRO-CLIMATE	
CO_2	CO_2 intensity
OUTDOOR WEATHER	
Raining	Rained or not
T-outer	Temperature (T) outside
RH-outer	Relative Humidity (RH) outside
*PAR-outer[2]	outside PAR sensor
PLANT TREATMENT	
LTG	Cyclic lighting (by the flowering bulbs)
Watering	Amount of irrigation
*pH [3]	pH of irrigation watering [4]
OTHER DATA	
PL	Weekly Plant Loads

We grouped the attributes according to their absolute correlation with the Brix, using thresholds of 0.3 and 0.5. Despite the fact that data under each category were highly correlated with each other, the selection of data did not give negative effects on the prediction performances. Neither did they have notable improvements in accuracy. Hence, we included only the best-performing model in the result section.

3 TIME SPANS OF THE DATA AND FEATURES

We archived hourly environmental records and weekly plant load data during the data collection period, as previewed in Table S2. We grouped the environmental information as rolling averages over every 7 days for the model training.

The plant load was recorded by calendar weeks, from the 12th week to the 42th week of 2021. As there was no finer time information available, the significance of plant load was analyzed in different branches of experiments when all the other environment information was also aggregated by calendar weeks.

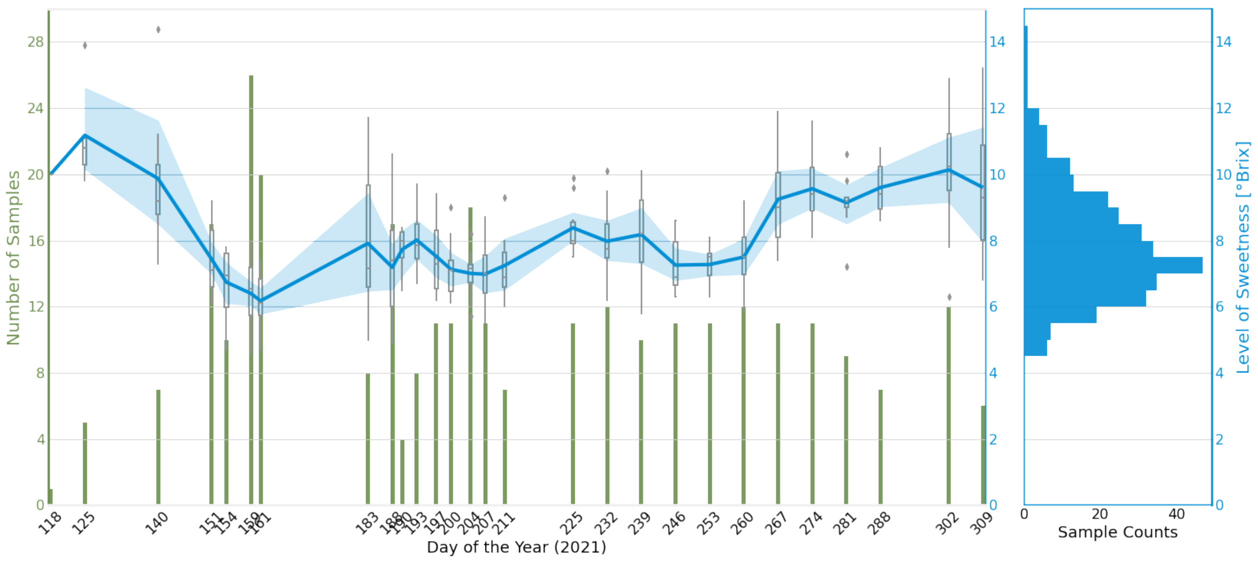

Figure S1. Statistics of the Brix measurements, grouped by the harvests. On the left, the x-axis indicates the day number of the harvests. The green y-axis presents the number of tested samples. The blue line and its contour indicate the averaged Brix value and the standard deviation (std.) of the measurements of the harvest respectively. The box plots illustrate the distribution of the measurement for the week. On the right, the histogram gives an overview of the distribution of all Brix measurements in 2021.

4 REGRESSION EXAMPLE

With the aggregated Brix information that was only predicted with environment records, we used the leave-one-out method to split the train and validation data. Figure S3 illustrates the prediction results from two selected models.

5 PARAMETER SELECTION OF REGRESSION MODELS

We tested four scales of regularizers (also named "alpha" in mathematical models) in the kernelized ridge regression (KRR), with three degrees of the polynomial kernel ("degree"). As is shown by Figure S4, the levels of fitness were illustrated by plotting the predicted Brix value with the first principal component of all the input features, which is all available environment records up to 21 days in advance. We finally decided to use both alpha=1 and alpha=10 in our experiment series.

6 DEEP LEARNING NEURAL NETWORKS

The architectures of the neural networks for the diverse purposes of machine learning are illustrated in S5. We trained two/three/four-layer CNNs from scratch for supervised learning (SL), with 12*12, 9*9, or 6*6 convolutional kernels. Each was connected with a two-layer multi-layer perception (MLP) which outputs the Brix value of each strawberry. When considering transfer learning for the SL, we replaced the CNNs trained from scratch with popular pre-trained models such as the *ResNet* and the *EfficientNet*. We used three-layer and four-layer CNNs to build the encoders and decoders for semi-supervised learning (SSL). We considered max-pooling layers of 2*2, 4*4 and/or 5*5 among the convolutional layers to reduce the feature volume of the latent space. The MLPs that mapped the latent space to the final output, i.e. the Brix values of strawberries, consisted of three or four fully-connected layers.

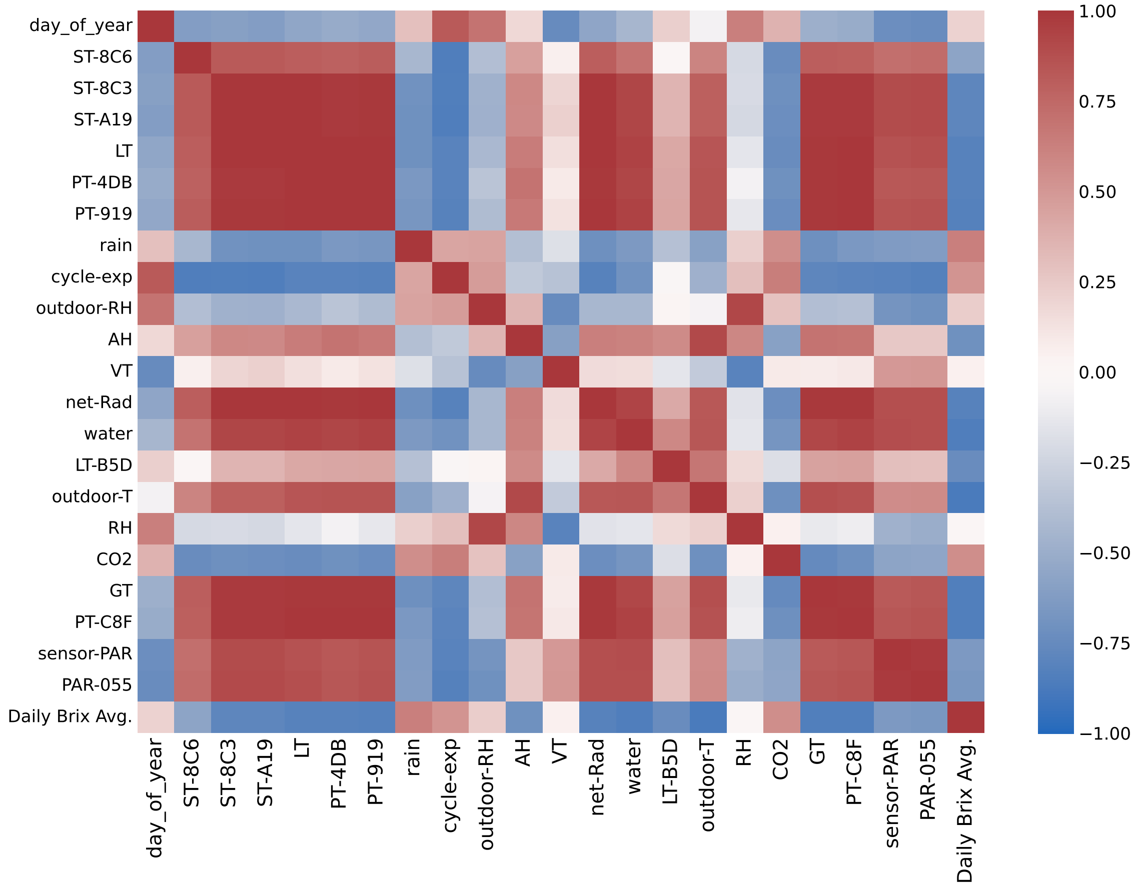

Figure S2. Correlations of each combination of features and the features with the daily averaged Brix. The color indicates the definition of each abbreviated name on the x-axis can be referred to Table S2. The heatmap gives the level of correlation.

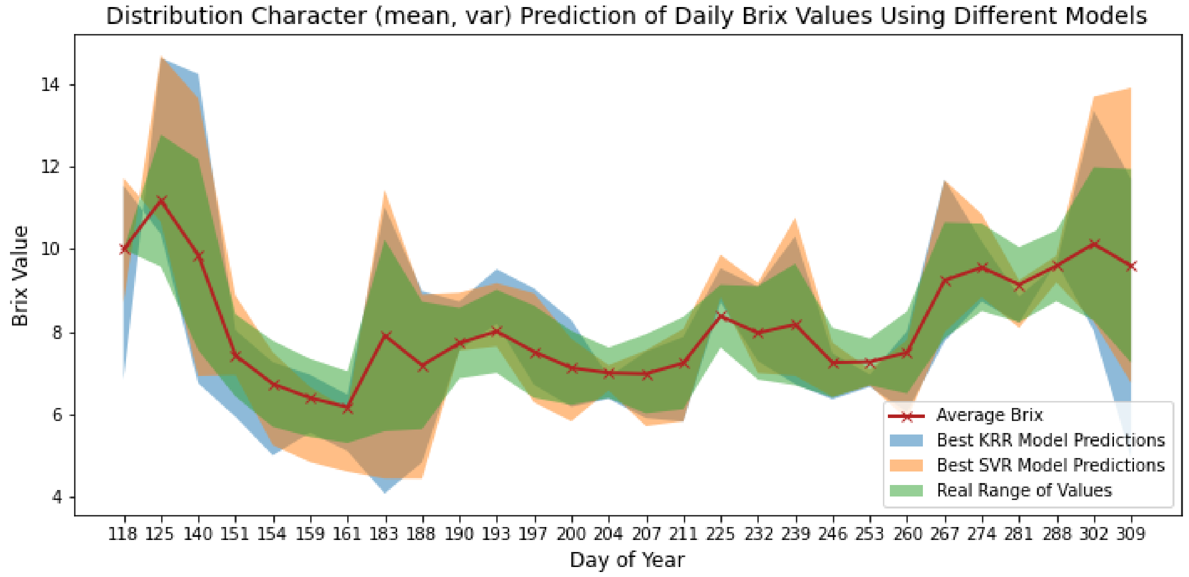

Figure S3. Brix distribution prediction over the measurement period. The distribution is illustrated by the (predicted) average and standard deviation of the Brix at the harvest day. The best model is selected by the minimum average RMSE over all data points, under various groups of feature selection.

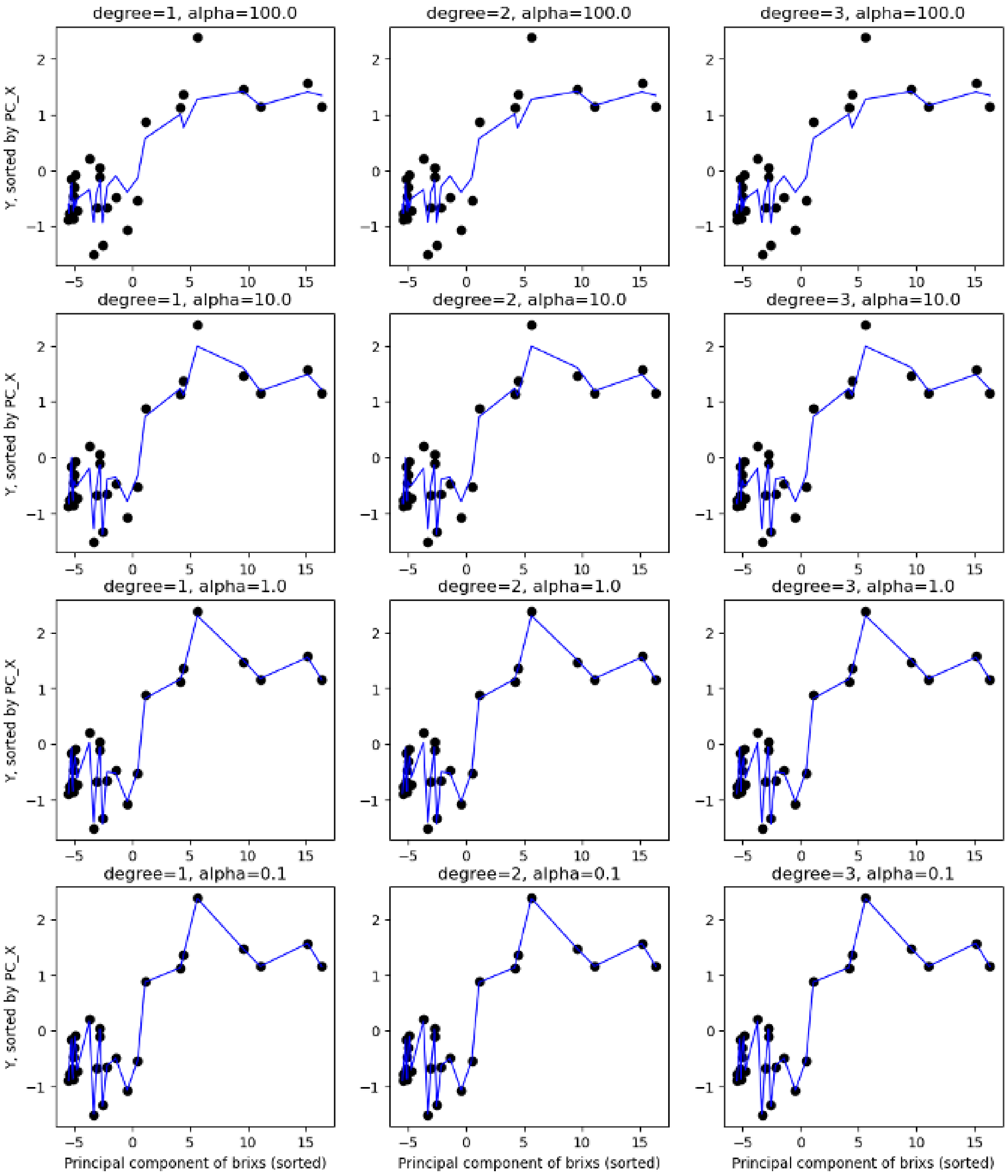

Figure S4. Parameter tests of the regularizer (alpha) and polynomial kernel (degree) in the kernelized ridge regression (KRR). We tested alpha=0.1,1,10,100 and polynomial degree=1,2,3 for deciding the final parameters. The influences from various alpha are discussed over rows, and the fitness under different polynomial degrees is compared within each row. The x-axis of each subplot indicates the value of the first principal component of the input feature. The y-axis of each subplot indicates the value of Brix. All the data points are sorted by the scale of the first principal component, so as to better present the influence from the regularizer.

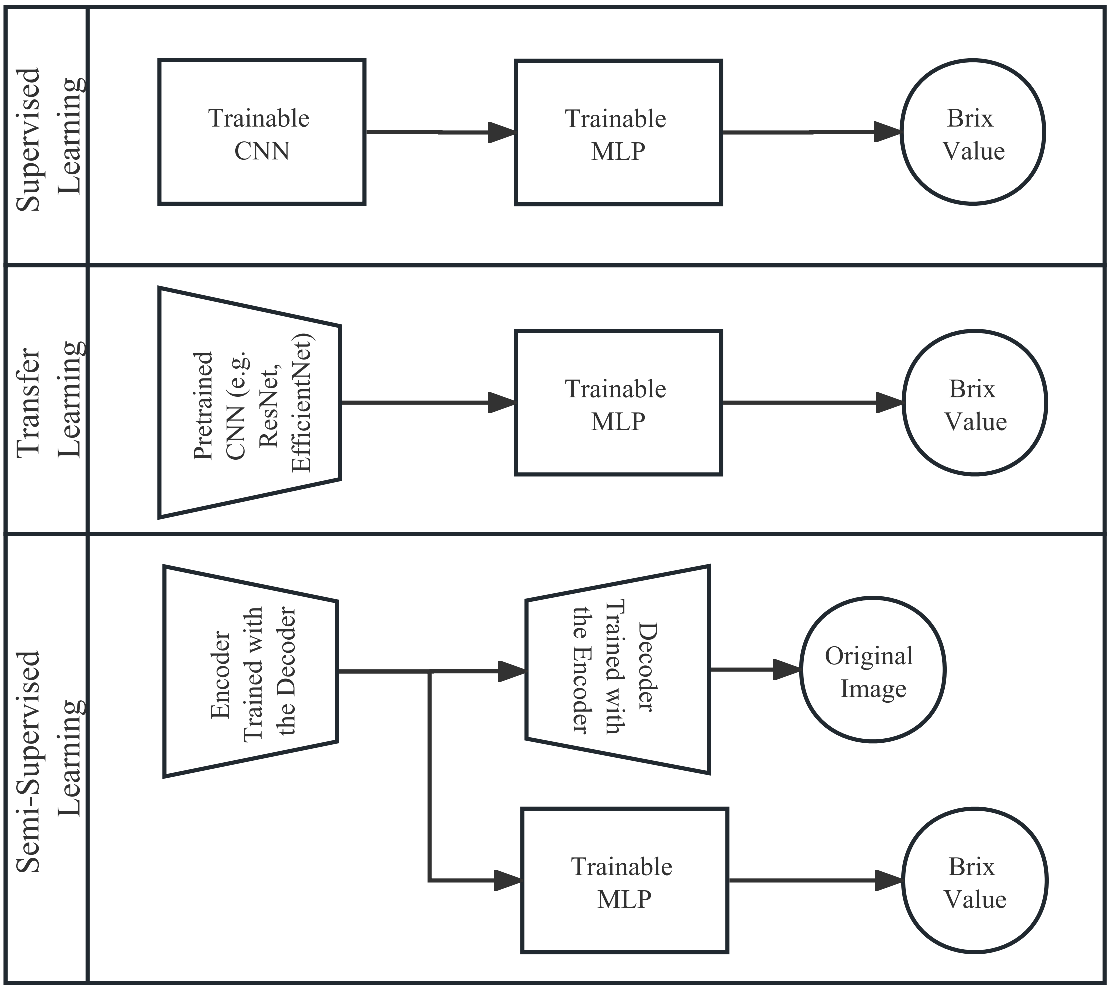

Figure S5. Illustration of the Learning Methods. The networks are presented in the form of functional blocks and the data flow among them. The rectangular boxes indicate the blocks that were trained by the image segments of the last available observation and the corresponding Brix of the strawberry. The trapezoidal boxes indicate that the blocks were trained by some other dataset: the pre-trained CNNs were trained by popular computer vision datasets such as the *ImageNet*; the encoders and decoders were trained by the entire image dataset, i.e. the observations of all monitored strawberries at most moments.
